# Unemployment and Job Search Behavior among People with Disabilities during the First Year of the COVID-19 Pandemic in Germany

**DOI:** 10.3390/ijerph20116036

**Published:** 2023-06-02

**Authors:** Karsten Ingmar Paul, Alfons Hollederer

**Affiliations:** 1 School of Business, Economics, and Society, Friedrich-Alexander Universität Erlangen-Nürnberg, 90403 Nürnberg, Germany; 2 Department of Social Work and Social Welfare, The Faculty of Human Sciences (FB 01), University of Kassel, 34127 Kassel, Germany; alfons.hollederer@uni-kassel.de

**Keywords:** disability, unemployment, COVID-19 pandemic, job search

## Abstract

Not much is known about how the COVID-19 pandemic affected the labor market experiences of people with disabilities. Since they constitute a generally disadvantaged group in the labor market, it is important to scrutinize whether their position has worsened during these difficult times and how they reacted with regard to their job search behavior. We therefore used data for the year 2020 from a large German panel (Panel Arbeitsmarkt und Soziale Sicherung, PASS), in order to scrutinize the prevalence of unemployment among people with disabilities (*N* = 739) during the first year of the COVID-19 pandemic. The factors that affected their unemployment status were also analyzed. The study found that people with legally recognized disabilities were more often unemployed than non-disabled people, even when controlling for possible confounding factors such as age, gender, or education. This effect was significant for severe disabilities and marginally significant for minor disabilities. Additionally, the type of disability affected the probability of being unemployed, with cardiovascular diseases, mental illnesses, and musculoskeletal disorders carrying a higher risk. In terms of job-seeking behavior, unemployed people with disabilities reported using some job search methods more frequently than their non-disabled counterparts. However, the intensity of the job search did not differ significantly between the two groups. Further differences were found when analyzing the reasons for abstinence from searching for a job, with unemployed people with disabilities primarily citing health-related factors (with a frequency of over 90%). In summary, health played a pivotal role in determining disabled people’s labor market experiences during the COVID-19 pandemic.

## 1. Introduction

With chronically low rates of employment [1], people with disabilities constitute a vulnerable group in terms of labor market participation and integration. During the pandemic, they also constituted a vulnerable group with regard to COVID-19. The severity of the coronavirus disease (COVID-19) as well as the mortality were higher among people with chronic diseases (which often lead to disability) than among people without chronic diseases [2]. In line with that, people with disabilities in several European countries reported in spring 2021 considerably more unmet healthcare needs than people without disabilities [3].

Furthermore, international studies have shown that people with a low socioeconomic status have a higher risk of contracting SARS-CoV-2 (severe acute respiratory syndrome coronavirus type 2) and that the course of the disease is more severe among them [4,5]. In accordance with this, in Germany unemployed people had an increased risk of hospitalization for COVID-19 when compared with employed people [6].

If one assumes that workers with disabilities had an elevated likelihood of becoming unemployed during the economic downturn that was triggered by the onset of the COVID-19 pandemic, then they represented a particularly vulnerable group, from both a health as well as a labor market perspective. Possibly, the two problems of disease vulnerability and labor market obstacles exacerbated each other. It is therefore imperative to scrutinize how prevalent unemployment was among people with disabilities during the COVID-19 pandemic and how they reacted to this unusual situation in terms of their job search behavior, which is an important predictor of finding employment [7].

Therefore, the main goal of the present investigation is to determine whether people with disabilities were affected more negatively with regard to their employment situation during the first year of the COVID-19 pandemic. Furthermore, since the intensity and methods of job seeking might have been influenced by the unprecedented situation of the COVID-19 pandemic, we also wanted to analyze how COVID-19 shaped disabled unemployed persons’ job search behavior and their attitudes towards looking for a job and entering employment in Germany.

The article is structured in the following way: We first present some general evidence concerning the typical labor market outcomes of people with disabilities, before we shortly review the still limited findings about their labor market outcomes during the COVID-19 pandemic. In the next section, we shortly review the few research findings on the job search behavior of people with disabilities. Then, research questions are formulated, mainly focusing on three major topics: (1) The extent of unemployment among people with disabilities during the pandemic; (2) the job search behavior of unemployed people with disabilities during the pandemic; and (3) COVID-19-related worries among people with and without disabilities. At the end of the introduction section, we describe the peculiarities of the German welfare system affecting people with disabilities and how Germany fared during the COVID-19 pandemic, in order to give some context for the empirical methods and results that follow.

The methods section first describes the data collection and then the methods used in the statistical analyses. The results section is structured in line with the three main topics described above. In the discussion, a summary and general interpretation of the main findings are given before the strengths and weaknesses of the study, as well as its practical implications, are discussed. Finally, a general conclusion closes the text.

### 1.1. Disability and Employment/Unemployment

Numerous studies have identified the negative effects of disability on labor market outcomes, such as employment and earnings [1]. Especially with regard to employment, studies in several countries have consistently shown differences in employment probabilities between disabled and non-disabled people, with disabled individuals being less frequently in employment when compared to their able-bodied counterparts (e.g., [8,9]). These differences in employment participation rates are of a large size and can only be explained to a limited degree by differences in productivity-related characteristics between both groups [10]. Other effects, such as prejudices against disabled people and the resulting wage discrimination that pushes some disabled people out of the labor market, also play an important role in explaining the observed differences in employment rates between people with and without disabilities [1,8,9]. Yet, the particularities of the job search behavior of disabled unemployed people have, to the best of our knowledge, not yet been systematically studied during the COVID-19 pandemic, although it could be substantially different from non-disabled people’s search behavior and might strongly influence labor market outcomes.

### 1.2. Disability and Employment/Unemployment during the COVID-19 Pandemic

Exactly what impact the COVID-19 pandemic had on the participation of people with disabilities in the labor market is not yet completely clear. A few early studies from the U.S. on this topic examined only small convenience samples. For example, during the initial phase of the COVID-19 pandemic, 20% of patients with multiple sclerosis reported losing their jobs due to the pandemic [11]. Individuals with developmental disabilities or intellectual disabilities for whom working in a home office was not an option were often placed on reduced hours or laid off [12]. Another study, with a larger sample, did find a significant drop in labor force participation among people with disabilities in the early stages of the COVID-19 pandemic, but this drop was not greater among people with disabilities compared to people without disabilities [13].

A clearer picture emerged through the use of data from the CPS, a monthly survey collected by the US Bureau of Labor Statistics that provides large samples covering the whole of the United States of America [14]. These analyses showed a significant drop in the employment rate of people with and without disabilities in early 2020. This drop was larger for people with disabilities (18.9%) than for people without disabilities (15.5%). The gap between the employment rates of disabled and non-disabled people then increased during the remainder of the first year of the COVID-19 pandemic, with women, predominantly black women with disabilities, being particularly strongly affected.

In the United Kingdom, on the other hand, an analysis using the Quarterly Labour Force Survey (QLFS), the largest representative household survey in the UK, could not identify relevant changes in the disability employment gap following the emergence of COVID-19 [15]. The proportion of unemployed workers slightly increased for both groups after the onset of the COVID-19 pandemic, but the gap between disabled and non-disabled workers did not change in any noteworthy way.

In summary, the picture is not yet clear with regard to what effects COVID-19 had on disabled and non-disabled people’s labor market experiences, particularly unemployment. However, it is important to know how well or how badly the disadvantaged group of people with disabilities fared during the COVD-19 pandemic, because should their labor market situation have worsened even more, some form of societal support would be urgently needed.

### 1.3. Disability and Job Search during COVID-19

In general, the role of stigmatization and prejudices during the job search has received some attention in the psychological literature. However, this was mainly focused on topics of race/ethnicity, age, and criminal record, while only a few studies have been performed on issues of disability [16]. Evidence regarding how the COVID-19 pandemic might have affected disabled people’s job search behavior, or their abstention from the job search, is even more scant and usually of an indirect nature.

In the United Kingdom, higher COVID-19-related economic and health risks were identified for disabled workers compared to non-disabled workers [15]. Among these risks were, for example, the likelihood to work in industries that experienced shutdowns, and working in occupations with greater proximity to others, increasing possible exposure to the disease. Given these risks and the elevated likelihood of a more severe course of the disease among people with disabilities, some might have temporarily resigned from actively searching for a new job during the COVID-19 pandemic.

A survey of people with disabilities in the midwestern USA from October 2020 to March 2021 found that people with disabilities encountered more challenges during the COVID-19 pandemic than people without disabilities [17]. A high percentage of them experienced employment changes or business shutdowns. Apart from pandemic-related reductions in business, the main reason for not working among people with disabilities were being sick or disabled (31.3%), or not wanting to work during the COVID-19 pandemic (22.9%).

It is important to scrutinize the differences in job search behavior of people with and without disabilities during the COVID-19 pandemic, because only when the commonalities and differences between the two groups are known does it become possible to tailor suitable interventions that accommodate the respective needs successfully and really help them in their job search efforts. Such knowledge is particularly important given that pandemics will occur in the future.

### 1.4. Research Questions/Goals

Building on the empirical evidence summarized above, our research goals were twofold: First, we wanted to know whether in Germany people with disabilities were more strongly affected by the COVID-19 pandemic with regard to their employment situation than people without disabilities. Such an effect was identified in the U.S., but it is not yet clear whether this phenomenon can also be found in countries with a more regulated labor market than the U.S. We were also interested in the role of specific aspects of disability, in particular the severity of disability and the type of disability, and their influence on the likelihood of unemployment.

Research question 1: Were disabled people more affected by unemployment during the early phases of the COVID-19 pandemic than non-disabled people?

Research question 2: Did severity and/or type of disability influence the likelihood of unemployment among disabled people during the early phases of the COVID-19 pandemic?

Furthermore, we wanted to know whether, and if so how, unemployed people with and without disabilities differed with regard to their job search behavior and related attitudes during the COVID-19 pandemic. We suspected that the threat of infection and the higher vulnerability of disabled people could have caused a higher reluctance to start a new job in the middle of such an unusual and seminal event as the COVID-19 pandemic, resulting in a weaker search activity. We also assumed that the reasons reported for not searching for a new job differed between unemployed people with and without disabilities, with health-related issues playing a more prominent role among the former group. It must also be considered that with certain previous illnesses the risk of a more severe course of the disease from COVID-19 increases sharply and that no vaccine was available during the observation period.

Research question 3: Are there differences in disabled people’s and non-disabled people’s job search behavior (with regard to intensity of job search and methods of job search)?

Research question 4: What reasons were reported by disabled and non-disabled unemployed persons for abstaining from job search during the early phases of the COVID-19 pandemic?

Finally, in order to obtain a more direct picture of the possible motivation behind the search behavior of people with and without disabilities during the COVID-19 pandemic, we also analyzed the answers to three questions specifically querying reactions to COVID-19. These questions asked whether the current COVID-19 crisis had triggered participants to worry about their current life situation, specifically with regard to (a) their own health, (b) the health of their family, and (c) their financial situation (the answering format was from 1 = “no worries” to 4 = “great worries”). We expected to find more worries, particularly more worries about one’s own health, among people with disabilities compared to people without disabilities.

Research question 5: Did people with and without disabilities differ with regard to the worries they experienced about COVID-19?

### 1.5. Contextualization: Disability, Unemployment and COVID-19 in Germany

In Germany, people are legally recognized as disabled if their physical function, mental ability, or mental health will most likely deviate from the typical health state for their age for more than six months in the prognosis and their participation in life in society is therefore impaired. The effects on participation in life and society are graded as the degree of disability in grades of ten (20–100) [18]. This grading is not primarily based on the causal diagnosis underlying the disability (e.g., cancer or depression), but on the manifestation of the disability and the functional limitations resulting from it. A degree of disability of 50 or more is defined as “severe” and implies special legal regulations, such as a stronger protection from dismissal and the possibility to retire early. However, the official recognition of disability is not automatically provided. Instead, disabled persons need to apply at specialized pension offices (“Versorgungsämter”) for this status, where every case is individually decided in accordance with criteria from the German Pension Medical Ordinance (“Versorgungsmedizinverordnung”) [19].

According to microcensus 2017, 10.2 million people were officially recognized as disabled in Germany, with 73% of them being severely disabled [20]. In 2019, 3.1 million people of working age, between 15 and 65, were recognized as severely disabled in Germany [18]. In the five years before the onset of the COVID-19 pandemic, unemployment rates were sinking, for people with disabilities as well as for people without disabilities, in Germany. Among the people with disabilities, unemployment rates declined from 13.4% in the year 2015 to 10.9% in 2019 in relation to the dependent labor force. The unemployment rate in the general population changed from 8.2% to 6.2% during the same time interval. Thus, while unemployment rates were generally sinking, the distance between people with and without disabilities remained stable [20].

The first cases of infection with SARS-CoV-2 in Germany occurred at the end of January 2020 [21]. Subsequently, the first year of the COVID-19 pandemic saw two major waves of infection, March to April 2020 and October 2020 to January 2021 [22]. On 24 March, the Bundestag declared an “epidemic situation of national concern” [23], and subsequently a range of measures was taken to contain the spread of infection, including travel restrictions, closures of schools, closures of non-essential businesses (including shops and restaurants), of sporting facilities, theatres, etc., social distancing regulations, mask-wearing regulations, and a ban on public gatherings [24]. In varying degrees of stringency (with more leniency during the summer months), these regulations were active until the end of the year 2020 and beyond.

The German economy reacted strongly to the pandemic and the countermeasures. The breakdown of world-wide supply chains [25], various lockdown measures, including factory closures and individual reactions of people leading to weak consumer demand, brought about a strong economic downturn, with the second quarter of 2020 registering the largest reduction in GDP since the year 1970 [26]. The downturn then eased in the third quarter, but for the whole year of 2020 a deep recession was registered for the German economy [27].

## 2. Materials and Methods

### 2.1. Data Collection

Data from the Panel Labor Market and Social Security (Panel Arbeitsmarkt und Soziale Sicherung, PASS) were used for this research project. The PASS is maintained by the Research Data Center (Forschungsdatenzentrum, FDZ) at the Institute for Employment Research (Institut für Arbeitsmarkt- und Berufsforschung, IAB) of the Federal Employment Agency (Bundesagentur für Arbeit, BA), Germany [28,29]. This panel is an annual survey of (1) persons from households receiving transfer benefits, and (2) persons from households in the general resident population.

With several thousand participants per wave, the panel also allows analyses for specific subgroups of the population, such as unemployed people with disabilities, because the test power remains sufficient. For the structure and the descriptives of the PASS panel, see [30].

A special feature of the PASS is that it explicitly asks whether a disability is officially recognized and what the official degree of disability is. In the German system, officially recognized disabilities can range in severity from 20 to 100, with higher degrees indicating greater severity.

The present study used data from wave 14 of the PASS. The start date of data collection for this wave was February 2020. However, the usual face-to-face interviewing had to be stopped in mid-March 2020 due to a nationwide COVID-19 lockdown, and data collection was switched to telephone interviews. In mid-May 2020, data collection could return to face-to-face interviews. The fieldwork was completed at the end of September 2020. The sample size was *N* = 9138 respondents.

For the present analysis, we used the subsamples of unemployed and employed people with and without officially recognized disabilities. The data were accessed via scientific use files of the Research Data Center (FDZ) of the Federal Employment Agency (BA) at the IAB [28].

### 2.2. Statistical Analyses

In order to scrutinize research question 1, we conducted a logistic regression analysis [31] with unemployment (vs. employment) as the dependent variable and disability status (officially registered as mildly or severely disabled or not disabled) as the predictor.

In order to learn more about the specific characteristics of disability that influenced whether a disabled person was unemployed or not (research question 2), we conducted a logistic regression analysis with unemployment (vs. employment) as the dependent variable and type of disability as the predictor.

Next, in order to test for possible differences in the job search behavior of disabled vs. non-disabled people (research question 3), we conducted a series of logistic regressions with disability status as the predictor and variables measuring seven different methods of job search behavior (plus an “other” category) as the dependent variables. Furthermore, among those unemployed persons who had not searched for a job, we conducted a series of additional logistic regressions with eleven possible reasons for not searching for a job, with disability status as the predictor and each of the reasons for not searching as the dependent variables.

Finally, in order to identify differences between people with and without disabilities with regard to their emotional reactions to COVID-19, we conducted *t*-tests for three Likert items measuring COVID-19-related worries. We also conducted a *t*-test with an index measuring job search intensity [32].

We recognized the possibility of third variables confounding our analyses. For example, age might be higher among disabled individuals than among non-disabled individuals, and at the same time, age discrimination might lead to higher levels of unemployment among older people compared to younger people, leading to a spurious association between disability and unemployment. Therefore, we used several variables that are likely to influence disability status or employment status as control variables. These were: (a) age, (b) birth country, (c) children in the household, (d) level of education, (e) vocational qualification, and (f) occupational status (manual worker vs. employee). The datasets were weighted before analysis in order to ascertain representativity of the German population [33] using the complex sample module of SPSS version 28.0.1.1.

## 3. Results

### 3.1. Characteristics of the Sample

We restricted our analyses to people with and without disabilities who were participants in the labor market, i.e., who were either employed at the moment of data collection or who were officially registered as unemployed. Furthermore, a small group of people who were not officially registered as disabled but had filed an application for being recognized as disabled were excluded due to the uncertain outcome of these applications.

The two groups of people with and without disabilities differed markedly with regard to several sociodemographic characteristics (see Table 1). In particular, persons with disabilities were significantly older, with an average age of *M* = 51.4 (SEM = 0.744) years, compared to *M* = 43.5 (SEM = 0.345) for the non-disabled group. The disabled group also included significantly fewer women (37.5%) than the non-disabled group (45.8%) and had significantly fewer children in their households (*M* = 0.5; SEM = 0.060) than the non-disabled counterparts (*M* = 0.8; SD = 0.029). Furthermore, a significantly larger proportion of the disabled study participants (90.5% vs. 81.6%) were born in Germany. They also had a significantly lower education, with 38.2% having attained only a secondary school diploma as their highest school leaving certificate, compared to 24.1% in the non-disabled group. Importantly, the rate of unemployment was almost twice as high in the group with disabilities (14.1%) compared to the group without disabilities (7.6%). Both groups had a high proportion of long-term unemployed people (78.8% vs. 69.7%). There were no significant differences for the rate of professional qualifications and the proportion of manual workers between the subgroups.

About half of the disabled participants (51.8%) were categorized as “severely disabled” in accordance with German social laws [34].

The partially strong differences between the disabled and non-disabled groups with regard to sociodemographic characteristics demonstrated the necessity to control the influence of these variables in the substantive analyses in order to avoid possible confounding influences.

### 3.2. Prevalence of Unemployment among People with Disabilities

When predicting the participants’ employment status (unemployed vs. employed) by their disability status, significant effects were found in the bivariate analysis as well as in the analysis with control variables (see Table 2). People with severe disabilities were significantly (*p* < 0.001 and *p* < 0.002) more often unemployed than non-disabled people in the first year of the COVID-19 pandemic. With an odds ratio of *OR* = 2.62 in the controlled analysis, this effect was of a substantial size. For people with minor disabilities, we also found an increased rate of unemployment in comparison to people without disabilities which was marginally significantly (*p* = 0.056 and *p* = 0.083). While this effect was weaker than the effect for severe disabilities (*OR* = 1.86), it was still strong enough to be of practical relevance.

A significant influence on employment status was also found for several control variables. A lower age (*OR* = 0.48; *p* < 0.001), a place of birth outside of Germany (*OR* = 0.55; *p* = 0.023), a small number of children in the household (*OR* = 0.56; *p* = 0.011), a low level of formal education (not more than secondary school certificate, *OR* = 2.22; *p* = 0.006), and a lack of professional qualifications (*OR* = 2.34; *p* = 0.002) were all associated with an increased probability of belonging to the group of the unemployed.

In summary, the risk of unemployment was significantly increased among disabled people, even when other important factors influencing the risk of unemployment were kept statistically constant. This was not only true for severely disabled people but could also be identified—at least as a marginally significant trend—for people with comparatively minor disabilities.

### 3.3. Type of Disability as Predictor of Unemployment

Next, we analyzed which specific types of health impairments were associated with unemployment (see Table 3). Significant effects were found for cardiovascular diseases (*OR* = 2.77; *p* = 0.009), mental illnesses (*OR* = 2.72; *p* = 0.005), and musculoskeletal disorder (*OR* = 2.03, *p* = 0.028). People with these kinds of health impairments had a significantly elevated risk of unemployment. The size of these effects was considerable, more than doubling the odds of being unemployed compared to people without these illnesses. We also identified a marginally significant trend for “other” forms of health impairment (*OR* = 2.76; *p* = 0.099), also increasing the risk of unemployment. Thus, in addition to the severity of a disability, the type of an individual’s health impairment also plays an important role with regard to their labor market outcomes: Some types of illness, in particular psychological disorders, musculoskeletal disorder, and cardiovascular disease, carry a higher risk of unemployment than other forms of disability.

In addition, we wanted to shed some light on the dynamics of the labor market for people with disabilities during the first year of the COVID-19 pandemic. In order to achieve this, we planned to analyze whether disabilities influenced the probability of losing one’s job during the early phases of the COVID-19 pandemic, and the likelihood of finding a new job during that time.

For the first question, we created a variable coding new cases of unemployment, i.e., people who became newly unemployed in 2020, as (1), and cases where the person had lost their job in 2019 or an earlier year and was still unemployed in 2020 as (0). A logistic regression predicting this variable (see Table 4) showed a significant effect for disability status, with people with minor disabilities showing a lower probability of belonging to the group of new entrants into unemployment than the group of people without disabilities. This effect could be identified in the uncontrolled analysis (*OR* = 0.20; *p* = 0.036), as well as in the controlled analysis (*OR* = 0.15; *p* = 0.016).

Unfortunately, the exit of severely disabled people from unemployment to employment decreased by 10.9% between 2019 and 2020 [20]. Their transition rate to employment was 3.2% in 2020. Regrettably, the second analysis, where we intended to test whether disability predicted changes from unemployment to employment during the first year of the COVID-19 pandemic did not yield interpretable results due to the very low number of participants who reported to have found a new job in 2020.

In summary, while people with disabilities were generally overrepresented among the unemployed in the first year of the COVID-19 pandemic in Germany, this was probably not the result of a high number of persons with a disability freshly losing their jobs at this time. On the contrary, new job losses appear to have been comparatively infrequent in this group at that time. The high prevalence of unemployment among people with disabilities could be the consequence of a “carry-over” effect from the years before the COVID-19 pandemic. Furthermore, the extremely low number of cases in the dataset who changed from unemployment to employment might indicate that hiring new employees slowed down considerably during the first year of the COVID-19 pandemic.

### 3.4. Job Search and COVID-19-Related Worries among Disabled and Non-Disabled Unemployed People

Next, we examined the job-seeking behavior of unemployed people with and without disabilities. We measured their general job search intensity with an index combining six Likert-type items, asking how often (from 1 = “less than weekly” to 4 = “daily”) an individual had recently performed six different types of job search (newspaper adds, job board of employment agency, online search, friends and relatives, private job agent, job agent of employment agency). For this measure, no significant difference between people with and without disabilities could be identified when the usual control variables were included in the analysis (*t* = 0.877; *p* = 0.381; see Table 7).

To obtain a more detailed picture, we also conducted additional analyses testing whether participants had used specific job search techniques recently (see Table 5). We found three significant differences between the groups. Unemployed people with disabilities reported looking for a new job through job advertisements in newspapers more often than unemployed people without disabilities (*OR* = 4.06; *p* = 0.014). They also reported using the internet for their job search significantly more frequently than unemployed people without disabilities (*OR* = 3.79; *p* = 0.028). Finally, more unemployed people with disabilities than without disabilities searched for jobs via “other” methods of job search (*OR* = 6.01; *p* = 0.022). No differences were found for the other comparisons (e.g., search through friends and relatives, private employment agencies, unsolicited applications, etc.). Overall, the picture of a relatively similar search behavior among unemployed people with and without disabilities emerged, with people with disabilities using three specific search methods more frequently.

We also analyzed how often unemployed people reported not to be searching for a job currently and what reasons they gave for this abstinence from searching. The percentage of people not looking for employment was significantly higher among the unemployed people with disabilities compared to their counterparts without disabilities (71.8% vs. 55.8%, *p* = 0.049, see Table 1). With regard to the reported reasons, some differences between unemployed people with vs. without disabilities emerged (see Table 6). One reason, i.e., “being in training” (*OR* = 0.19; *p* = 0.042) was given significantly more often by unemployed people without disabilities. Additionally, “being in a work program” (*OR* = 0.20; *p* = 0.057) emerged as a marginally significant trend in the same direction. On the other hand, two arguments were cited significantly more often by unemployed people with disabilities, namely, “health reasons” (*OR* = 6.05; *p* < 0.001), and “my financial situation would not improve” (if a job were found) (*OR* = 2.62; *p* = 0.033). The very large odds ratio of *OR* = 6.05 for health reasons indicates that this difference between the two groups was very strong. Among the disabled unemployed, 92.6% agreed with this reason for not looking for a new job, compared to only 53.5% of the non-disabled.

### 3.5. COVID-19-Related Worries among People with and without Disabilities

Finally, in order to answer research question 5, we also analyzed the answers to three Likert-type items measuring participants’ worries with regard to COVID-19 (see Table 7). We found a significant effect for COVID-19 worries concerning one’s own health (*t* = −4.452; *p* < 0.001). Disabled people experienced this kind of worries more often than non-disabled people did. Furthermore, there was also a significant (*t* = −2.470; *p* = 0.014) difference with regard to COVID-19-related worries about the health of relatives. Again, unemployed people with disabilities reported more of these worries than non-disabled people did. No significant difference was found for COVID-19 worries about one’s financial situation (*t* = 0.644; *p* = 0.520).

**Table 7:** Comparisons between unemployed persons with vs. without disability on job search intensity and COVID-19-related worries.

Dependent Variable	With Disability M (SEM)	*95% CI*	Without Disability M (SEM)	*95% CI*	*t*	df	*p*
Job search intensity	2.25 (0.203)	1.85; 2.65	2.43 (0.162)	2.11; 2.75	0.877	380	0.381
COVID-19 worries about own health	3.07 (0.110)	2.85; 3.28	2.62 (0.073)	2.47; 2.76	−4.452	2377	0.001
COVID-19 worries about health of relatives	3.45 (0.088)	3.28; 3.63	3.23 (0.060)	3.11; 3.35	−2.470	2369	0.014
COVID-19 worries about financial situation	2.39 (0.116)	2.16; 2.62	2.46 (0.077)	2.31; 2.61	0.644	2371	0.520

Note: Each line shows the estimates of a separate weighted linear regression with controls; results for the control variables not displayed here for reasons of space. Sample size, *N* = 444–2446. M = mean value; SEM = standard error; *95% CI* = 95% confidence interval for mean; t = students *t*-test statistic; df = degrees of freedom; *p* = significance level for *t*.

## 4. Discussion

The presented results demonstrate that people with disabilities in Germany had a higher risk of unemployment during the first year of the COVID-19 pandemic than people without disabilities. However, they did not have an elevated risk of becoming new entrants to unemployment during this first year. Thus, findings from U.S. studies hinting at people with disabilities being particularly strongly hit by negative employment effects of the COVID-19 pandemic [11,12,14] could not be replicated here. The present results are more in line with other European findings showing similar employment trends for people with and without disabilities during the early phases of the pandemic [15].

Research on the job search behavior of unemployed people with disabilities is very rare [35]. Nevertheless, it is unlikely that differences in job-seeking behavior are the reason for the higher prevalence of unemployment among people with disabilities, because our analyses have shown search behavior to be similar for disabled and non-disabled unemployed people. Indeed, with regard to some specific techniques of job search, disabled unemployed people were more active than non-disabled people. Employer prejudices and discrimination, as have been described in the international literature [1], might also be important reasons for this phenomenon in Germany, as in other countries. However, these effects were not specifically studied here.

Furthermore, the results showed the likelihood of unemployment to depend on the type of disability. These results are in good agreement with the general assumption in the job search literature that people with different kinds of disabilities experience different levels of stigmatization and different types of obstacles during the job search process [36]. However, experimental findings showing that hiring-related attitudes against people with mental disabilities are more negative than attitudes against people with physical disabilities [37] were only partly endorsed by our results: The odds of being unemployed were equally strong for people with two specific types of physical disabilities, as for people with a mental disability. Specifically, the employment outcomes for musculoskeletal disorders and cardiovascular disease were as bad as the outcomes for mental illnesses.

Additionally, there were some interesting group differences in reported reasons for not seeking a new job. Among disabled unemployed persons, health reasons were clearly dominant, with more than 90% reporting this reason for their search abstinence, while only about half gave this reason among the people without disabilities. This demonstrates that the topic of health is of massive importance for employment-related decisions among people with disabilities. The other reason given more often by people with disabilities was of a financial nature (“financial situation would not improve”). This might be a consequence of the generally lower level of income workers with a disability receive in Germany compared to workers without a disability [38].

Furthermore, two other reasons were reported less frequently if a disability was present compared to people without disabilities. These reasons (“in training” and “in work program”) hint at an ongoing process of (re)integration into the labor market that people without disabilities experience but where people with disabilities appear to be missing out.

In line with the findings on health problems as reasons for not seeking employment, COVID-19-related worries, particularly with regard to health, were articulated considerably more often by disabled people than by non-disabled people. Apparently, the issue of health is of crucial importance for people with disabilities when it comes to deciding whether or not to look for a job, and in times of a severe health threat such as COVID-19, health-related considerations become the dominant driving force for the decision whether to look for a new job or not.

### 4.1. Strengths and Limitations

The main strength of the present study lies in the use of a large sample, which also covers the group of unemployed people with disabilities and thus makes detailed analyses of their job search behavior possible in the first place. As such, it is the only study we know of that was able to look at specific details of job search behavior in a group that is often neglected in labor market research.

Another strength of the study could be seen in the method of measurement of disability. We did not use a subjective self-assessment of disability or work capacity, as is typically used in other studies on the topic in labor market research [1]. Instead, the German state’s categorization system was used, which assigns every individual a specific degree of disability, with the scoring being performed by health authorities, not the individual. There is a good chance that this method helped to reduce problems of measurement error and a possible justification bias, i.e., the tendency to over-report disability to justify one’s unemployment [39,40].

A possible problem could be seen in the temporary change in the survey methods from face-to-face interviews to telephone interviews due to COVID-19 pandemic restrictions limiting direct social contacts. Studies have shown the survey mode to influence responses. In particular, telephone interviews appear to be prone to heightened social desirability and reduced reporting accuracy compared to other methods such as face-to-face interviews [41,42]. However, the survey mode could only have confounded our results if we had compared different time points within the year 2020 with each other (e.g., spring vs. fall), which was not the case.

Another limitation is that the data comes only from the first year of the COVID-19 pandemic as data for later years was not yet available at the moment of the writing of this paper. It is conceivable that certain changes in job search behavior only occurred later during the course of the COVID-19 pandemic and were therefore not yet detectable with the available data. In addition, only a few items scrutinizing the phenomenon of a global health emergency caused by a new virus (SARS-CoV-2 virus) were included in the survey and could thus be used here.

With regard to the health restrictions that prevent people with disabilities from looking for a job, it would be desirable to find out what adjustments disabled people would like to see on the employer side, particularly with regard to infectious diseases. Corresponding items need to be developed and included in future surveys.

### 4.2. Implications

An earlier study from Australia found that disability did not influence job search intensity, but was negatively associated with the likelihood of engaging in job search [43]. Our results generally endorse these earlier findings (although three specific job search techniques were used more frequently by people with disabilities in the present study). Thus, it is highly important to identify the reasons that unemployed people with disabilities have for not actively searching for a job. Our results are helpful in this regard, by showing that during the time of a severe health threat such as COVID-19, health considerations become the clearly dominant factor in determining this decision. Should further research in post-pandemic circumstances replicate these findings, practitioners trying to support unemployed people with disabilities in their job search would have gained an important piece of information. It would be clear then that the job opportunity being safe, and the absence of any danger of further impediments to the health of the disabled job seeker, is probably the single most important aspect of every potential job, and that this issue needs to be scrutinized very thoroughly. This does not mean that other obstacles that disabled people searching for a job experience, such as transportation problems [44], should be ignored. However, they are probably less decisive than health considerations.

An important avenue for further research could thus be the development of specialized intervention programs for unemployed people with disabilities. An important aspect of such a program might be the stabilization of participants’ job search self-efficacy, which has been reported to be fragile among job seekers with disabilities [45]. Furthermore, involving job seekers who have been successful in their job hunt could lead to valuable advice for the construction of such specialized intervention programs [46].

## 5. Conclusions

The present findings showed that health considerations were of high importance for unemployed people with disabilities when they decided whether to search for a job during the COVID-19 pandemic or not. Furthermore, workers’ health status to a large degree determined their employment status, i.e., whether they had a job or were unemployed. In the current time of severe labor shortages, the need for companies and organizations to hire people with disabilities is likely to increase. This pragmatic consideration alone makes it inevitable that protection from health hazards is sufficiently guaranteed. Furthermore, Germany has ratified the UN convention on the rights of persons with disabilities [47], explicitly committing itself to “protect the rights of persons with disabilities, on an equal basis with others, to just and favorable conditions of work, including (…) safe and healthy working conditions“ (Article 27b). Given the growing proportion of people with disabilities among working-age adults [48], it is now highly urgent to implement these commitments in practice.

## Figures and Tables

**Table 1:** Demographic characteristics of disabled and non-disabled employed and unemployed participants.

	Disabled Participants		Non-Disabled Participants		
	*n*	*%*	*n*	%	*p*
Severely disabled (degree of disability ≥ 50)	285/551	51.8	---	---	---
Age—50 years or older	372/556	66.9	1925/5366	35.9	0.001
Age—mean (SEM)		51.4 (0.744)		43.5 (0.345)	0.001
Female gender	209/556	37.5	2457/5367	45.8	0.048
Country of birth: Germany	503/556	90.5	4375/5361	81.6	0.009
Low education (max. secondary school)	212/556	38.2	1294/5366	24.1	0.001
No professional qualifications	76/557	13.6	689/5361	12.9	0.787
Manual worker	127/340	37.4	870/2674	32.5	0.355
Children in household? (yes)	158/556	28.5	2636/5366	49.1	0.001
Number of children in household—mean (SEM)		0.5 (0.060)		0.8 (0.029)	0.001
Currently unemployed	79/556	14.1	410/5366	7.6	0.001
Among unemployed: Currently long-term unemployed (≥1 year)	232/295	78.8	1081/1551	69.7	0.178
Among unemployed: Not searching for a job	178/247	71.8	740/1326	55.8	0.049
Among not searching for a job: Health reasons	176/190	92.5	368/794	46.4	0.001

Note. *n* = sample size; *p* = significance level for group comparison; % = proportion of disabled or non-diabled participants whom belong to the respective category (e.g., 50 years or older); Age range: 16–65; Range of degree of disability: 2–100. Education: max. secondary school computed as “Hauptschule”, “Sonderschule” or “no school certificate”; Dataset was weighted before analyses.

**Table 2:** Disability as a predictor of unemployment—logistic regressions.

	Analysis	B	*SE_B_*	Wald *χ*^2^	*p*	*OR*	*95% CI*
**Disability**	Without controls						
Severe disability		0.817	0.244	11.224	0.001	2.264	1.40; 3.65
Minor disability		0.479	0.251	3.646	0.056	1.615	0.99; 2.64
Intercept		1.195	0.340	125.680	0.000	---	--
**Disability**	Control variables included						
Severe disability		0.963	0.303	10.090	0.002	2.620	1.45; 4.75
Minor disability		0.620	0.357	3.010	0.083	1.859	0.92; 3.75
*Demographic characteristics* **Age**—50 years or older							
		−0.725	0.221	10.725	0.001	0.484	0.31; 0.75
**Gender**—Female		0.046	0.225	0.0423	0.837	0.955	0.61; 1.49
**Country of birth**—Germany		−0.601	0.265	5.147	0.023	0.549	0.33; 0.92
*Household structure* **Children in household**							
		−0.572	0.224	6.517	0.011	0.564	0.36; 0.88
*Education* **Highest school leaving certificate** Secondary or special school							
					
		0.795	0.287	7.655	0.006	2.215	1.26; 3.89
**Professional qualification** No professional qualification							
		0.849	0.270	9.883	0.002	2.338	1.38; 3.97
*Socio-economic variables* **Last occupational position** Manual worker							
					
		0.346	0.263	1.738	0.188	1.413	0.85; 2.37
Intercept		−0.0064	0.573	10.625	0.001	---	---

Note. *B* = unstandardized regression weight; *SE_B_* = standard error of *B*; Wald *χ*^2^ = test statistic; *p* = significance level for Wald *χ*^2^; *OR* = odds ratio; *95% CI* = 95% confidence interval for odds ratio. Reference category for disability: no disability; Reference category for age: up to 49 years; Reference category for gender: male; Reference category for country of birth: any other country than Germany; Reference category for children: no children living in household; Reference category for highest school leaving certificate: certificate above secondary school; Reference category for no professional qualification: some kind of professional qualification; Reference category for last occupational position: other position, e.g., clerical worker.

**Table 3:** Type of disability as a predictor of unemployment—logistic regression.

	B	*SE_B_*	Wald *χ*^2^	*p*	*OR*	*95% CI*
Type of disability:						
Cardiovascular disease	1.019	0.392	6.754	0.009	2.771	1.28; 5.98
Musculoskeletal disorder	0.710	0.323	4.818	0.028	2.034	1.08; 3.84
Visual or hearing impairment	−0.361	0.384	0.888	0.346	0.697	0.33; 1.48
Cancer	−0.625	0.453	1.907	0.168	0.535	0.22; 1.30
Metabolic disease	−0.329	0.407	0.652	0.420	0.72	0.32; 1.60
Allergy	0.270	0.364	0.551	0.458	1.311	0.64; 2.68
Internal disease	0.336	0.355	0.895	0.344	1.399	0.70; 2.81
Mental illness	1.002	0.353	8.067	0.005	2.724	1.36; 5.45
Other type of disability	1.015	0.614	2.734	0.099	2.759	0.83; 9.20
Control variables:						
Age—50 years or older	−0.898	0.337	77.122	0.008	0.407	0.21; 0.79
Female gender	−0.260	0.328	0.630	0.428	1.297	0.68; 2.47
Country of birth: Germany	−1.680	0.492	11.653	0.001	0.186	0.07; 0.49
Children living in household	−1.081	0.384	7.917	0.005	0.339	0.16; 0.72
Max. secondary school	0.652	0.353	3.407	0.065	1.920	0.96; 3.84
No professional qualifications	1.050	0.418	6.319	0.012	2.858	1.26; 6.49
Manual worker	0.094	0.360	0.069	0.793	1.099	0.54; 2.23
Constant	−0.820	1.235	0.583	0.445	--	--

Note. Sample size: *N* = 1032. Explained variance: Nagelkerke *R*^2^ = 0.342. Dataset was weighted before analysis. *B* = unstandardized regression weight; *SE_B_* = standard error of *B*; Wald *χ*^2^ = test statistic; *p* = significance level for Wald *χ*^2^; *OR* = odds ratio; *95% CI* = 95% confidence interval for odds ratio. Reference category for disability: no disability; Reference category for age: up to 49 years; Reference category for gender: male; Reference category for country of birth: any other country than Germany; Reference category for children: no children living in household; Reference category for highest school leaving certificate: certificate above secondary school; Reference category for no professional qualification: some kind of professional qualification; Reference category for manual worker: other position, e.g., clerical worker.

**Table 4:** Disability as a predictor of a recent (in the year 2020) entrance to unemployment compared to older entrances—logistic regressions.

	Analysis	B	*SE_B_*	Wald *χ*^2^	*p*	*OR*	*95% CI*
**Disability**	Without controls						
Severe disability		−0.430	0.672	0.409	0.523	0.651	0.17; 2.43
Minor disability		−1.602	0.765	4.384	0.036	0.202	0.05; 0.90
Intercept		3.603	0.996	28.457	0.001	--	--
**Disability**	Control variables included						
Severe disability		−0.360	0.749	0.231	0.631	0.698	0.16; 3.03
Minor disability		−1.926	0.802	5.768	0.016	0.146	0.03; 0.70
Age—50 years or older		−0.251	0.469	0.287	0.593	0.778	0.31; 1.95
Female gender		−0.820	0.508	2.601	0.107	2.270	0.84; 6.16
Country of birth: Germany		0.321	0.452	0.504	0.478	1.378	0.57; 3.35
Children living in household		0.243	0.456	0.284	0.594	1.275	0.52; 3.12
Max. secondary school		0.233	0.494	0.222	0.638	1.262	0.48; 3.33
No professional qualification		−0.581	0.516	1.267	0.261	0.559	0.20; 1.54
Manual worker		−0.808	0.469	2.964	0.085	0.446	0.18; 1.12
Intercept		4.752	1.309	19.397	0.001	--	--

Note. Sample size: first analysis, *N* = 1835; second analysis, *N* = 1162. Explained variance: first analysis, Nagelkerke *R*^2^ = 0.019; second analysis, *R*^2^ = 0.115. Datasets were weighted before analyses. *B* = unstandardized regression weight; *SE_B_* = standard error of *B*; Wald *χ*^2^ = test statistic; *p* = significance level for Wald *χ*^2^; *OR* = odds ratio; *95% CI* = 95% confidence interval for odds ratio. Reference category for disability: no disability; Reference category for age: up to 49 years; Reference category for gender: male; Reference category for country of birth: any other country than Germany; Reference category for children: no children living in household; Reference category for highest school leaving certificate: certificate above secondary school; Reference category for no professional qualification: some kind of professional qualification; Reference category for manual worker: other position, e.g., clerical worker.

**Table 5:** Disability as a predictor of job search methods among unemployed people—logistic regressions.

Dependent Variables: Job Search Method	B	*SE_B_*	Wald *χ*^2^	*p*	*OR*	*95% CI*	*R* ^2^	*Frequency among Disabled*	*Frequency among Non-Disabled*
Newspaper ads	1.401	0.567	6.099	0.014	4.060	1.33; 12.39	0.259	68.9%	36.5%
Online job board of employment agency	0.186	0.621	0.089	0.765	1.204	0.36; 4.08	0.221	57.8%	58.4%
Internet search	1.333	0.604	4.871	0.028	3.793	1.16; 12.44	0.393	82.9%	71.3%
Search via friends and relatives	0.470	0.615	0.585	0.445	1.600	0.48; 5.36	0.139	60.8%	64.4%
Job agent in employment agency	0.752	0.669	1.265	0.261	2.122	0.57; 7.91	0.202	43.9%	36.9%
Privat job agent	−0.229	0.992	0.053	0.818	0.795	0.11; 5.59	0.084	6.4%	8.3%
Initiative job search	−1.050	1.170	0.804	0.370	0.350	0.04; 3.50	0.141	4.7%	9.6%
Other methods	1.793	0.779	5.297	0.022	6.006	1.30; 27.78	0.553	10.7%	0.9%

Note: Each line shows the results of a separate logistic regression with controls; for reasons of space, results for the control variables and the constant are not displayed here; percentages were estimated without controls. *B* = unstandardized regression weight; *SE_B_* = standard error of B; Wald *χ*^2^ = test statistic; *p* = significance level for Wald *χ*^2^; *OR* = odds ratio; *95% CI* = 95% confidence interval for odds ratio; *R*^2^ = explained variance according to Nagelkerke *R*^2^; datasets were weighted before analysis.

**Table 6:** Disability as a predictor of different reasons for not searching for a job among unemployed people—logistic regressions.

Dependent Variables: Reasons for Not Searching for a Job	B	*SE_B_*	Wald *χ*^2^	*p*	*OR*	*95% CI*	*R* ^2^	*Frequency among Disabled*	*Frequency among Non-Disabled*
Household income sufficient	0.536	0.524	1.043	0.307	1.708	0.61; 4.78	0.255	36.9%	13.9%
Financial situation would not improve	0.964	0.452	4.556	0.033	2.622	1.08; 6.36	0.082	47.1%	25.6%
Already found new job	−0.089	0.757	0.014	0.907	0.915	0.21; 4.05	0.361	7.2%	16.5%
Health reasons	1.800	0.494	13.257	0.001	6.048	2.29; 15.97	0.320	92.6%	53.5%
Childcare/other care	−0.698	0.815	0.733	0.392	0.498	0.10; 2.47	0.506	8.6%	38.1%
Too few jobs available	−0.401	0.544	0.545	0.461	0.670	0.23; 1.95	0.108	19.9%	24.4%
Former job search unsuccessful	0.108	0.502	0.046	0.830	1.114	0.42; 2.98	0.157	29.3%	21.8%
Retirement	0.522	0.604	0.749	0.387	1.686	0.52; 5.52	0.413	28.4%	6.0%
Training	−1.676	0.824	4.135	0.042	0.187	0.04; 0.94	0.380	1.1%	15.3%
Work program	−1.608	0.844	3.629	0.057	0.200	0.04; 1.05	0.252	4.4%	24.6%
Corona restrictions	0.259	0.611	0.180	0.672	1.296	0.39; 4.32	0.090	44.5%	36.1%

Note: Each line shows the results of a separate logistic regression with controls; for reasons of space, results for the control variables and the constant are not displayed here; percentages were estimated without controls. *B* = unstandardized regression weight; *SE_B_* = standard error of *B*; Wald *χ*^2^ = test statistic; *p* = significance level for Wald *χ*^2^; *OR* = odds ratio; *95% CI* = 95% confidence interval for odds ratio; *R*^2^ = explained variance according to Nagelkerke *R*^2^; datasets were weighted before analysis.

## Data Availability

Data access was provided via a scientific use file supplied by the Research Data Centre (FDZ) of the German Federal Employment Agency (BA) at the Institute for Employment Research (IAB).

## Data

Bähr et al. Panel Study Labor Market and Social Security (PASS)—Version 0619 v3”. Research Data Centre of the Federal Employment Agency (BA) at the Institute for Employment Research (IAB), Nuremberg, Germany, 2021. DOI: 10.5164/IAB.PASS-SUF0619.de.en.v3.

## Data Documentation

Berg et al. Codebuch und Dokumentation des Panel ‘Arbeitsmarkt und soziale Sicherung’ (PASS)—Datenreport Welle 13. FDZ Datenreport, 12/2020 (de), Nuremberg, Germany, 2020. DOI: 10.5164/IAB.FDZD.2012.de.v1.
